# Patient-Related Risk Factors for Postoperative Infection After Cholecystectomy

**DOI:** 10.1007/s00268-017-4029-0

**Published:** 2017-06-20

**Authors:** Gona Jaafar, Folke Hammarqvist, Lars Enochsson, Gabriel Sandblom

**Affiliations:** 1 Department of Clinical Sciences, Intervention and Technology (CLINTEC), Centre for Digestive Diseases, Karolinska Institute, (Gastrocentrum) Karolinska University Hospital, 141 86 Stockholm, Sweden; 2 P03, Karolinska University Hospital-Solna, 17176 Stockholm, Sweden; 3 Department of Surgical and Perioperative Sciences, Umeå University, Umeå, Sweden

## Abstract

**Background:**

The impact of patient-related risk factors on the incidence of postoperative infection after cholecystectomy is relatively unknown.

**Aim:**

The aim of this study was to explore potential patient-related risk factors for surgical site infection (SSI) and septicaemia following cholecystectomy.

**Materials and methods:**

All cholecystectomies registered in the Swedish national population-based register for Gallstone Surgery and Endoscopic Retrograde Cholangiopancreatography (GallRiks) 2006–2014 were identified. The study cohort was cross-matched with the Swedish National Patient Register in order to obtain data on patient history and postoperative infections. Simple and multiple logistic regression analyses were performed in order to assess the impact of various comorbidities on the risk for SSI and septicaemia.

**Results:**

A total of 94,557 procedures were registered. A SSI was seen following 5300 procedures (5.6%), and septicaemia following 661 procedures (0.7%). There was a significantly increased risk for SSI in patients with connective tissue disease (odds ratio [OR] 1.404, 95% confidence interval [CI] 1.208–1.633), complicated diabetes (OR 1.435, CI 1.205–1.708), uncomplicated diabetes (OR 1.391, CI 1.264–1.530), chronic kidney disease (OR 1.788, CI 1.458–2.192), cirrhosis (OR 1.764, CI 1.268–2.454) and obesity (OR 1.630, CI 1.475–1.802). There was a significantly higher risk for septicaemia in patients with chronic kidney disease (OR 3.065, CI 2.120–4.430) or cirrhosis (OR 5.016, CI 3.019–8.336).

**Conclusion and discussion:**

Certain comorbidities have an impact on the risk for postoperative infection after cholecystectomy, especially SSI. This should be taken into account when planning the procedure and when deciding on prophylactic antibiotic treatment.

## Background

Cholecystectomy is one of the most common abdominal surgical procedures in the world. In most cases, it is performed without major risk for serious complication, although bile leakage and intraoperative contamination may lead to surgical site infection (SSI). Nevertheless, all risks must be taken into account when surgery is considered. The incidence of SSI after cholecystectomy was found to be 2.4% in a meta-analysis of studies on perioperative antibiotics in laparoscopic cholecystectomy [1]. In elective cholecystectomy, there is a low incidence of postoperative infection so the routine use of antibiotic prophylaxis (AP) is not recommended [2–4]. After cholecystectomy for acute cholecystitis, the risk is significantly higher [5]. The lack of internationally accepted guidelines concerning AP in cholecystectomy has led to divergent routines regarding the use of AP in surgery for acute cholecystitis. A better understanding of risk factors for postoperative infection may help in gaining consensus on antibiotic guidelines.

Risk factors related to the procedure itself are well-known and have been thoroughly studied [6] [7]. The risk for postoperative infection related to comorbidity, on the other hand, is not as well understood. Postoperative infection prolongs time in hospital and has a negative effect on recovery and healthcare costs. AP has not been shown to decrease the risk for postoperative infection in elective cholecystectomy [5], and a register-based study on AP in acute cholecystectomy, found that it did not reduce the risk even when adjusting for confounders [1].

There may be other factors, not yet identified, that influence the risk for postoperative infection. Furthermore, we are not aware of any measure effective in preventing infection. A recent study showed, for instance, that abdominal drainage does not prevent intra-abdominal complications after laparoscopic cholecystectomy for acute cholecystitis [8].

## Aim

The aim of this study was to investigate procedure- and patient-related risk factors associated with postoperative infection. The outcomes were surgical site infection (SSI) including intra-abdominal abscess, and septicaemia including cholangitis.

## Materials and method

The study population was obtained from the GallRiks. GallRiks is a Swedish national quality register for gallstone surgery and ERCP, which began in May 2005 [9]. Since then, it has gradually reached a national coverage above 85%. It is continuously validated, showing high accuracy and completeness of data [10]. By the end of 2014, more than 90,000 cholecystectomies had been registered in the GallRiks. The patient records of all patients registered in the GallRiks are reviewed 30 days postoperatively by a local coordinator in order to detect and register all postoperative complications. In any case where deviation from the expected course is noted, the coordinator either registers this as a complication or confers with the local surgeon responsible as to its nature.

Variables registered include indication for surgery (uncomplicated gallstone disease or complications secondary to gallstone disease such as cholecystitis, pancreatitis or obstructive jaundice due to common bile duct stone). Patients with uncomplicated gallstone disease are predominantly operated electively, whereas those with complications often undergo acute surgery.

The Swedish National Inpatient Register (IPR) is a part of the National Patient Register (NPR). It has had national coverage since 1987. All diagnoses are coded according to the International Classification of Diseases code (ICD code). In the NPR, 99% of all discharges are registered, including ICD codes for all relevant diagnoses. In a validation study, the correctness of the entries was found to be 85–95% [11].

All diagnoses in the postoperative course were retrieved from GallRiks as well as NPR based on ICD code registered by physician responsible for the discharge. SSI was defined as infection necessitating antibiotic treatment or percutaneous drainage in GallRiks, and/or ICD code T81.4 from NPR. Septicaemia was defined as cholangitis in Gallriks and/or cholangitis (ICD code K83.0) and sepsis (ICD code A40 and A41) in NPR. We preferred the term septicaemia since the criteria included positive blood culture, not necessarily including a systemic inflammatory response to the septicaemia [12].

All procedures performed from 2006–2014 were included in the study. Data on procedure-related risk factors and relevant patient-related risk factors were obtained from the GallRiks. Patient-related risk factors and comorbidities were extracted from Swedish IPR. Cross-checking between the GallRiks and the International Patient Register (IPR) was performed.

Simple and multiple logistic regression analyses were performed in order to analyse risk factors for SSI and septicaemia. The covariates retrieved from the GallRiks were age (younger or older than 70 years), gender, ASA (1 vs >1), indication for cholecystectomy (elective due to biliary colic as sole indication versus acute due to cholecystitis, pancreatitis or obstructive jaundice), method of approach (laparoscopic or open and converted), operation time (less or more than 120 min), antibiotic treatment and accidental gallbladder perforation. The covariates extracted from the IPR were: history of connective tissue disease (ICD codes M05–M06, M31.5, M32–M34, M35.1, M35.3 and M36.0); diabetes mellitus (ICD codes E10-E14); chronic kidney disease(ICD codes N03.2–N03.7, N05.2–N05.7, N18, N19, I12.0, I13.1, Z49.0–Z49.2, Z94.0, Z99.2); liver cirrhosis (ICD codes K70.3, K71.7, K74, I85); immunodeficiency (ICD codes D80–D89); and obesity (ICD code E86), according to the ICD codes retrieved from NPR.

The study was approved by the Regional Research Ethics Committee, Stockholm, Sweden, and data were processed without entering the patients’ files.

## Result

A total of 94,557 procedures were registered during the study period. The outcomes of the simple logistic analyses are shown in Table 1. OR with a 95% confidence interval for SSI were significant for all covariates apart from immunodeficiency (Table 1). Regarding septicaemia, ORs were significant with 95% confidence interval for all covariates except accidental gallbladder perforation, immunodeficiency and obesity (Table 1).

Multivariate analyses with SSI and septicaemia as outcome variables were performed, with patient-related comorbidities as covariates, adjusted for age, gender, ASA, indication for surgery, method of approach, operation time, antibiotic treatment and accidental gallbladder perforation. Regarding SSI, the OR was significantly increased for connective tissue disease, complicated and uncomplicated diabetes, chronic kidney disease, cirrhosis and obesity but not for immunodeficiency (Table 2). The risk for septicaemia was significantly increased in cirrhosis and chronic kidney diseases but not with the other comorbidities (Table 2).

**Table 1:** Simple logistic regression analyses for SSI and septicaemia with covariates indicated

Variable	Surgical site infection					Septicaemia				
	Simple logistic regression analyses for SSI					Simple logistic regression analyses for septicaemia				
	*N*	%	*P* value	Odds ratio	CI	*N*	%	*P* value	Odds ratio	CI
Age, >70 years versus <70 years	1375/12,725	10.8	<0.001	2.405	2.254–2.565	232/12,725	1.8	<0.001	3.524	3.000–4.139
Gender, male versus female	2255/31,068	7.3	<0.001	1.554	1.469–1.643	341/31,068	1.1	<0.001	2.191	1.880–2.553
ASA >1 versus 1	3493/45,385	7.7	<0.001	2.186	2.062–2.317	501/45,385	1.1	<0.001	3.419	2.860–4.087
Indication for surgery, gallstone pain or complications of gallstone disease	3216/39,876	8.1	<0.001	2.214	2.092–2.343	514/39,876	1.3	<0.001	4.844	4.031–5.822
Open approach, including conversion from laparoscopic to open or laparoscopic	1868/13,450	13.9	<0.001	3.705	3.490–3.934	309/13,450	2.3	<0.001	5.705	4.881–6.669
Op. time >120 min	1985/22,711	8.7	<0.001	1.991	1.879–2.110	301/22,711	1.3	<0.001	2.756	2.360–3.219
Antibiotic treatment	2632/31,025	8.5	<0.001	2.130	2.015–2.253	438/31,025	1.4	<0.001	4.319	3.661–5.095
Accidental gallbladder perforation	1868/27,490	6.8	<0.001	1.364	1.287–1.446	201/27,490	0.7	0.226	1.109	0.938–1.311
Connective tissue disease	216/2035	10.6	<0.001	2.044	1.770–2.360	38/2035	1.9	<0.001	2.807	2.017–3.907
Complicated diabetes	166/1269	13.1	<0.001	2.587	2.192–3.052	27/1269	2.1	<0.001	3.177	2.153–4.689
Uncomplicated diabetes	608/5283	11.5	<0.001	2.347	2.146–2.567	97/5283	1.8	<0.001	2.943	2.368–3.657
Chronic kidney disease	123/788	15.6	<0.001	3.168	2.608–3.848	33/788	4.2	<0.001	6.483	4.536–9.268
Cirrhosis	44/345	12.8	<0.001	2.476	1.802–3.402	17/345	4.9	<0.001	7.531	4.597–12.338
Immunodeficiency	28/489	5.7	0.904	1.024	0.698–1.501	4/489	0.8	0.752	1.173	0.437–3.146
Obesity	507/6173	8.2	<0.001	1.562	1.420–1.719	46/6173	0.7	0.653	1.072	0.793–1.447

**Table 2:** Multiple logistic regression analyses for patient-related risk factors after adjustment for other confounders

Conditions	Multiple logistic analyses for SSI			Multiple logistic analyses for septicaemia		
	*P* value	Odds ratio	CI	*P* value	Odds ratio	CI
Connective tissue disease	<0.001	1.404	1.208–1.633	0.004	1.656	1.170–2.343
Complicated diabetes	<0.001	1.435	1.205–1.708	0.158	1.345	0.891–2.029
Uncomplicated diabetes	<0.001	1.391	1.264–1.530	0.023	1.313	1.039–1.660
Chronic kidney disease	<0.001	1.788	1.458–2.192	<0.001	3.065	2.120–4.430
Cirrhosis	0.001	1.764	1.268–2.454	<0.001	5.016	3.019–8.336
Immunodeficiency	0.468	0.864	0.582–1.282	0.916	1.055	0.391–2.847
Obesitiy	<0.001	1.630	1.475–1.802	0.261	1.196	0.875–1.635

Septicaemia within 30 days postoperatively was registered in the IPR following 538 procedures (0.6%). Postoperative septic cholangitis was registered following 175 procedures (0.2%) in the GallRiks. For 63 procedures, sepsis and/or septic cholangitis were registered in the GallRiks as well as in the IPR. Surgical site infection or infection requiring antibiotics were registered in the GallRiks following 4835 of the procedures (5.2%). Wound infections within 30 days postoperatively were registered after 1532 of the procedures (1.6%). Surgical site infection or infection requiring antibiotics were registered in the GallRiks as well as in the IPR following 1136 of the procedures.

## Discussion

Although the comorbidity conditions studied in the present cohort had only a moderate impact on the risk for postoperative infection, the study indicates that certain patient-related factors should be taken into account when planning surgery for gallstone disease. In the case of patient-related risk factors, the risk for infection may, to some extent, be reduced by AP. Although surgical technique is of crucial relevance, more so than any other preventive measure, risk factors related to the patient *per se* should not be neglected. We are aware of patient-related risk factors prior to surgery, and these must be taken into account when planning the procedure and optimising patient safety. By taking these factors into account, and using tailored AP and careful surgical technique, the risk for postoperative infection may be decreased.

In the present study, connective tissue disease, complicated and uncomplicated diabetes, chronic kidney disease, cirrhosis and obesity, when adjusted for gender, age, ASA >1, operation indication, open approach, prolonged operation time, antibiotic treatment and gallbladder perforation, were found to increase the risk for SSI. An increased risk for septicaemia was associated with chronic kidney disease and cirrhosis.

The rate of complications requiring surgical or percutaneous reintervetion following laparoscopic cholecystectomy is approximately 10%. In a study on 4359 patients undergoing laparoscopic cholecystectomy, it was found that patients with cholecystitis have an increased risk for developing bile leakage, intra-abdominal abscess and pneumonia [7]. This was also confirmed in the present study where indications for acute operation such as complicated gallstone disease increased the risk for both SSI and septicaemia. Older patients (>70 years) also have an increased risk for postoperative infection, which is in accordance with the results in the present study.

In elective laparoscopic cholecystectomy, there is generally low risk for postoperative infection. The routine use of AP was therefore not recommended in a Cochrane report from 2010 [13]. However, for patients with high risk factors for postoperative infection, AP should be considered even if a complicated procedure is not anticipated.

The decision to perform cholecystectomy may be a confounder for a preoperatively foreseen risk for surgical complications, including surgical site infection [9]. In the present study, open surgery or conversion to open surgery increased the risk for SSI and septicaemia by a factor of three and four, respectively.

Poorly controlled diabetes with postoperative hyperglycaemia has been reported to increase the risk for postoperative wound infection [14]. In the present study, both complicated and uncomplicated diabetes were associated with more than double the risk for SSI and septicaemia.

There are well-known risk factors for postoperative infection. The present study shows the risk for SSI to be increased by factors of three and five in patients with chronic kidney disease and cirrhosis, respectively. The indication for surgery should therefore be weighed against the risk of performing gallstone surgery on these patient groups. In cases where surgery is unavoidable, AP should be considered and meticulous care must be taken to avoid spillage of bile and haemorrhage.

Patient comorbidity, in particular liver cirrhosis, chronic kidney disease and obesity, should be taken into account when deciding on antibiotic prophylaxis. On the other hand, overuse of AP must also be avoided. With the increasing spread of antibiotic resistance, great efforts should be taken to avoid unnecessary use of AP. Culture-directed antibiotic treatment decreases the chance of inadequate AP in acute cholecystectomy, thereby contributing to the fight against antibiotic resistance.

The present study was based on the IPR as a source of data on previous medical history. Even if the register has a high completeness, not all relevant diagnoses may have been reported, e.g. immunodeficiency was defined from the ICD codes, without uniform criteria for immunological testing. Immunodeficiency would probably have been found to have a stronger impact if more data had been available. Furthermore, even if the GallRiks is continuously validated, there may be under-reporting of postoperative infections.

In conclusion, the present study shows that comorbidity, in particular liver cirrhosis, kidney disease and obesity, is important risk factor for SSI and septicaemia following cholecystectomy. Even if the risk factors investigated in the present study did not have as much impact as that seen with other risk factors, such as the presence of acute cholecystitis, conversion to open surgery, perioperative bleeding or bile leakage and patient-related factors should be taken into account when planning the procedure and when deciding on AP.
